# Targeting Proteinase Activated Receptor-4 Reduces Mechanonociception During the Acute Inflammatory Phase but not the Chronic Neuropathic Phase of Osteoarthritis in Rats

**DOI:** 10.3389/fphar.2021.756632

**Published:** 2021-12-22

**Authors:** Melissa S. O’Brien, Jason J. McDougall

**Affiliations:** Departments of Pharmacology and Anaesthesia, Pain Management and Perioperative Medicine, Dalhousie University, Halifax, NS, Canada

**Keywords:** arthritis, animal model, protease (proteinase)-activated receptor, pain, inflammation, electrophysiology, neuropathy

## Abstract

Serine proteases are elevated in arthritic joints where they can cleave protease activated receptors (PARs) to modulate pain and inflammation. Activation of protease-activated receptor 4 (PAR4) has been implicated in inflammatory joint pain. Whether PAR4 is involved in osteoarthritis (OA) pain has not yet been explored. The aim of this study was to compare the role of PAR4 in modulating early versus late stage OA pain using two models of OA *viz.* monoiodoacetate (MIA) and medial meniscal transection (MMT). G-ratio calculation and electron microscopy analysis revealed saphenous nerve demyelination and structural damage during late stage but not early OA in both models. Using immunohistochemistry, neuronal expression of PAR4 was higher in early versus late OA. Systemic administration of the PAR4 antagonist pepducin P4pal10 reduced both secondary allodynia (von Frey hair algesiometry) and joint nociceptor firing (single unit recordings) in MMT and MIA animals compared to vehicle-treated animals in early OA. The PAR4 antagonist was ineffective at altering pain or joint afferent firing in post-inflammatory OA. During the acute phase of the models, joint inflammation as determined by laser speckle contrast analysis and intravital microscopy could be partially blocked by pepducin P4pal10. Compared to late-stage disease, inflammatory cytokines were elevated in early MIA and MMT rats. These findings suggest that PAR4 may be a viable target to treat the pain of early onset OA or during episodic inflammatory flares.

## 1 Introduction

Osteoarthritis (OA) is a whole joint disease characterized by various levels of tissue degradation, inflammation, neuropathy, and pain. The aetiology and pathogenesis of OA is multifarious amongst patient such that it is no longer considered a single disease of common origin (Deveza et al., 2019). Known promoters of OA include joint malalignment, obesity, age, sex, and an inapt healing response to joint trauma. The molecular mediators responsible for joint destruction and pain are diverse; however, one family of mediators that transcends pathophysiology and symptom are the serine proteinases. In addition to their catabolic properties, serine proteinases can signal pain and inflammation in joints by a unique receptor pathway (McDougall and Muley, 2015).

Proteinase activated receptors (PARs) are a class of G protein-coupled receptors consisting of four known members (PAR1-PAR4) which are substrates of serine proteinases. Enzymatic cleavage of the N-terminus reveals a tethered ligand that interacts with the extracellular docking site on the same receptor leading to receptor activation. PAR1 is present on afferent nerve terminals and can be activated by the enzymatic action of thrombin. Intra-plantar injection of thrombin increases the nociceptive threshold to noxious mechanical and thermal stimuli (Asfaha et al., 2002; Kawabata et al., 2002). In synovial joints, PAR1 is expressed in the cartilage, synovium, and bone (Morris et al., 1996) where it can be either protective (Jastrzebski et al., 2019) or pro-arthritic (Yang et al., 2005). Arthritic mice lacking PAR1 exhibit enhanced pain sensitivity suggesting that PAR1 can confer anti-nociception in joints (Martin et al., 2009). PAR2 is expressed throughout the joint including the synovium (Kelso et al., 2007) and neurones (Russell et al., 2012). PAR2 activation in a murine model of OA has been found to mediate pain, inflammation, and the development of joint neuropathy (Muley et al., 2016; Muley et al., 2017). Using both PAR2 knockout mice and a PAR2 antagonist, pain and inflammation are significantly reduced, and OA-induced nerve damage is abolished (Muley et al., 2017). Another study using a post-traumatic model of OA also found that PAR2 is involved in pain sensitivity as well as osteophyte formation (Huesa et al., 2016). The serine proteases matriptase and neutrophil elastase are present in knees where they can activate PAR2 leading to joint pain and inflammation (Muley et al., 2017; Ariffin et al., 2021). PAR4 activation in the joint is similarly pro-inflammatory and pro-algesic. In mice, intraarticular injection of the synthetic PAR4 activating peptide AYPGKF-NH2 increases pain behaviour and causes synovial hyperaemia (McDougall et al., 2009). When administered to normal joints, the activating peptide causes sensitization of joint nociceptors via a mechanism that involves mast cell degranulation and bradykinin B2 receptor engagement (Russell et al., 2010). It is currently unknown whether PAR4 activation also contributes to joint neuropathy and neuropathic pain.

Animal models of OA facilitate the examination of disease pathogensis and enable drug target validation for pain control. The monoiodoacetate (MIA) model of OA pain is commonly used in rodents whereby intra-articular injection of the toxin inhibits chondrocyte glycolysis leading to cartilage degeneration and pain. MIA injection causes a biphasic pain response over 14-days that is characterised by early joint inflammation and end-stage neuropathy (Muley et al., 2017; Philpott et al., 2017). Medial meniscus transection (MMT) models post-traumatic OA (PTOA) where the surgical insult produces a slow deterioration of the joint resulting in OA-like lesions and degenerative joint pain (Bendele, 2002; Janusz et al., 2002). Joint afferent sensitisation and behavioural pain oberserved at the end-stage of this model is partially mediated by peripheral neuropathy; however, the early pain phenotype in this model has not been fully characterised (Bove et al., 2006; O'Brien and McDougall, 2020). While these models differ in pathogenesis, they provide a useful platform to test potential analgesic drugs under different disease conditions.

The aim of this study was to compare PAR4 blockade on joint pain and inflammation during the early and late stages of experimental OA.

## 2 Methods

### 2.1 Animals

Male Wistar rats (250–395 g, Charles River, Canada) were housed in pairs in ventilated racks on a 12 h light:dark cycle. Cages were supplemented with animal enrichment. Following arrival at the animal care facility, all rats were permitted at least 1 week to acclimate to their environment prior to entry into the study. Standard lab chow and water were provided *ad libitum* throughout the study. All experimental protocols were pre-approved by the Dalhousie University Committee on the Use of Laboratory Animals, which acts in accordance with the standards put forth by the Canadian Council for Animal Care.

### 2.2 Medial Meniscus Transection Model

Animals were deeply anaesthetized (2.5–4% isoflurane; 100% oxygen at 0.8 L/min) which was confirmed by unresponsiveness to noxious toe pinch and absence of a corneal blink reflex. The right (ipsilateral) knee joint was shaved and swabbed with chlorohexidine, ethanol, and betadine. A small incision was made in the skin covering the medial aspect of the knee and the medial collateral ligament was exposed by blunt dissection before being transected at midsubstance. The medial meniscus was visulaised and subsequently transected in the midsubstance. Palpation of the meniscus ensured transection prior to closure of the skin with 5–0 polypropylene suture. All animals were treated postoperatively with 1.2 mg/kg buprenorphine in a sustained release formulation that lasted 48–72 h. Days 7 (early phase) and 28–30 (late phase) post-surgery were used as the experimental timepoints in this study and were compared to naïve control animals.

### 2.3 Monoiodoacetate (MIA) Model of OA

The right (ipsilateral) knee joint of deeply anaesthetized animals (2.5–4% isoflurane; 100% oxygen at 0.8 L/min) was shaved and swabbed with chlorohexidine, ethanol, and betadine. 3 mg of MIA (in 50 μL saline) was introduced into the knee cavity via a Hamilton syringe inserted between the femoral condyles and the tibial plateau. The knee was then manually extended and flexed for 30 s to disperse the solution throughout the joint. Days 3 (early phase) and 14 (late phase) post-MIA induction were used as the experimental timepoints in this study and were compared to naïve control animals.

**FIGURE 1:**
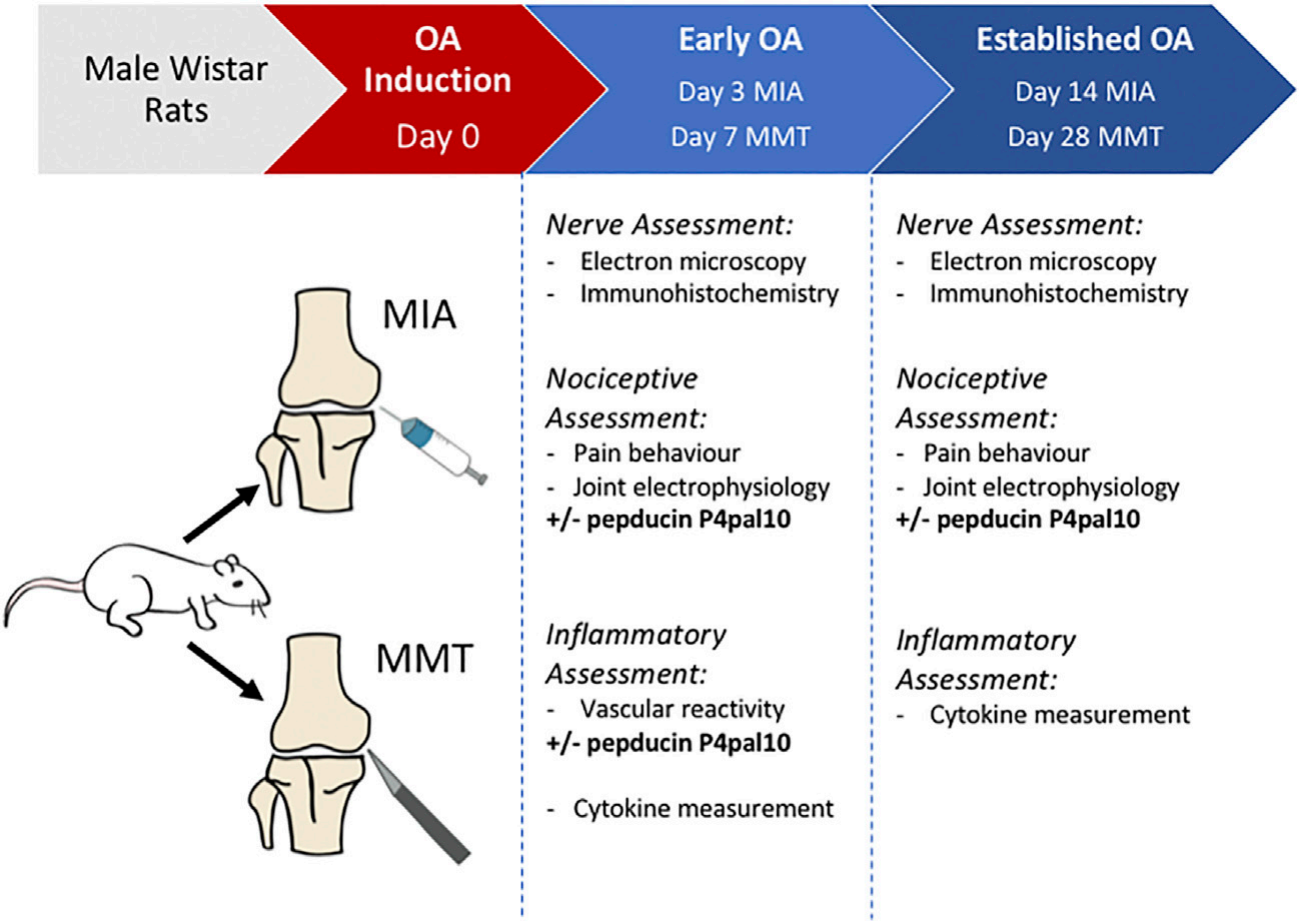
Experimental timeline. Experimental OA is induced in male Wistar rats using either intra-articular injection of sodium monoiodoacetate (MIA), or medial meniscal transection (MMT) surgery. *In vivo* and *ex vivo* assessments of OA animals are undertaken during an early stage (day 3 MIA, day 7 MMT) or an established/late stage (day 14 MIA, day 28 MMT) of the disease. Naïve animals were used as a control.

Figure 1 depicts the timeline of the study.

### 2.4 Pain Behavior

#### 2.4.1 von Frey Hair Algesiometry

Mechanical secondary allodynia was measured using a modified version of Dixon’s up-down method as previously described [6]. Animals were placed in a Perspex chamber with a metal mesh floor and allowed to acclimate for approximately 10 min prior to testing. Graded von Frey hairs were applied to the plantar surface of the ipsilateral hindpaw for up to 3 s. The hairs were applied in an ascending manner such that higher bending force hairs were applied following the absence of a positive response (withdrawal, lick, or shake of the paw) and lower force hairs were applied following a positive response to the filament. A cut off of 15 g bending force was enforced in this test.

#### 2.4.2 Dynamic Incapacitance

Animals were placed in a dynamic incapacitance chamber with a pressure sensitive floor (model BIO-DWB-AUTO-R, Bioseb, France). Hindlimb weight bearing was tracked in freely moving animals and recorded over a 3 min period. Weight borne on the ipsilateral hindpaw was calculated as a percentage of the total weight borne on both hindlimbs.

### 2.5 Electrophysiological Recordings of Joint Mechanosensory Afferents

Animals were deeply anaesthetized using urethane (25% solution; 2 g/kg i.p.), placed in a supine position on a thermostatically-controlled heating blanket, and a longitudinal incision was made in the neck. The trachea was exposed and cannulated to allow artificial ventilation with a Harvard rodent respiratory pump (100% O_2_ stroke volume: 2.5 ml; respiratory frequency: 52 breaths/min) (Harvard Apparatus, MA, United States). The jugular vein was cannulated for administration of the muscle relaxant gallamine triethiodide (50 mg/kg) which eliminated hind limb neuromuscular activity. The skin was incised along the medial aspect of the hindlimb, reflected, and secured to a metal “O” ring to create a pouch which was filled with warm mineral oil. The medial articular branch of the saphenous nerve was isolated and transected in the inguinal region to prevent spinal reflexes. The perineurium was removed from the distal nerve stump. Fine neurofilaments were isolated by teasing and then placed on a platinum recording electrode to measure single unit electrical activity. To identify a joint afferent fibre and its receptive field, the knee joint was gently probed with a blunt glass rod. Special care was taken to apply mechanical stimulation of the knee joint and not the hip or ankle joints. The hip joint was immobilized by applying a clamp to the femur which was attached to a stereotaxic frame. The right hind paw was placed in a shoe-like holder that immobilized the ankle joint. The shoe was connected to a force transducer and torque meter (Data Track 244-1-R, Intertechnology, Canada) such that upon movement a standardized amount of rotational force could be applied to the knee joint.

The mechanical threshold of each joint afferent was assessed by manually rotating the knee until the unit started to fire. Nerve activity in response to normal movement was highly variable therefore only noxious movements were tested. A noxious movement involved hyper-rotating the knee against soft tissue resistance to an extent that was outside the normal working range of the knee, but not severe enough to cause soft tissue injury. In an alert animal this would be considered a painful movement. The number of action potentials elicited during this noxious rotation were later counted offline. Three sets of noxious rotations, each lasting 5 s, were applied 5 min apart as a baseline measurement of afferent activity. The PAR4 antagonist pepducin P4pal10 (300 μg/kg) or vehicle (saline) was administered intraperitoneally (i.p.) and joint mechanosensitivity was assessed for an additional 60 min. The percentage change in afferent activity before and after administration of pepducin P4pal10 or vehicle was calculated off-line using Spike2 software (Cambridge Electronic Design, Cambridge, United Kingdom). The dose of pepducin P4pal10 was based on previously published findings (McDougall et al., 2009).

At the end of the experiment, the conduction velocity of the fibres was determined by electrically stimulating the receptive field with silver bipolar stimulating electrodes (0.6 Hz, 2 ms pulse width, 1–5 V) and measuring the latency of the evoked action potential.

### 2.6 Inflammation Measures

Animals were deeply anaesthetized using urethane (25% solution; 2 g/kg i.p.) 7 days post- MMT surgery and 3 days post MIA-injection. Rats were placed in a supine position and a longitudinal incision was made in the neck to expose the trachea which was cannulated to permit unrestricted breathing. Mean arterial pressure (MAP) was monitored via cannulation of the right carotid artery and recorded throughout the experiment.

#### 2.6.1 Intravital Microscopy

The ipsilateral knee was exposed by surgically removing the overlying skin and superficial fascia. Physiological buffer (37 ± 1°C) was continuously perfused over the exposed joint.

Intravital microscopy (IVM) was used to assess leukocyte trafficking within the microcirculation of the knee joint, as described previously (Andruski et al., 2008). Rhodamine 6G (0.05%), which stains nucleated cells, was administered via the jugular vein in order to visualize the circulating leukocytes *in vivo*. The synovial microcirculation was visualized under 590 nm fluorescent light using a Leica DM2500 microscope and images were captured using a Leica DFC 3000 camera (Leica Microsystems Inc., Ontario, Canada). Straight, unbranched post-capillary venules were identified, and three one-minute videos of fluorescently-labelled leukocytes were captured for each time point. Following baseline measurements, 300 μg/kg pepducin P4pal10 or vehicle (saline) was administered i.p. and recordings were continued for 120 min.

Two measures of leukocyte-endothelial interactions were used to assess articular inflammation: 1) the number of rolling leukocytes to pass an arbitrary line perpendicular to the venule in 1 min, and 2) the number of adherent leukocytes within a 100 μm portion of the venule. Rolling leukocytes were defined as positively stained blood cells travelling slower than the surrounding blood flow, and adherent leukocytes were defined as positively stained cells that remained attached to the endothelium for a minimum of 30 s.

#### 2.6.2 Laser Speckle Contrast Analysis

Joint blood flow was measured by laser speckle contrast analysis (LASCA) using a PeriCam PSI System (Perimed Inc., Ardmore, PA, United States). Recordings of the exposed knee joint were taken over 1 min at a working distance of 10 cm with a frame capture rate of 25 images per second. Using dedicated software (PIMSoft, Version 1.5.4.8078), images were averaged to generate 1 perfusion image per second. Following baseline measurements, 300 μg/kg pepducin P4pal10 or vehicle (saline) was administered i.p. and recordings were continued for 120 min.

At the end of the experiment rats were euthanized and a dead scan of the knee was taken*.* This “biological zero” value which corresponds to tissue Brownian motion was subtracted from all previous measurements. Images were analyzed offline where mean blood perfusion in a defined region of interest approximating the knee joint was calculated. To account for differences in blood perfusion based on the MAP of the animal, vascular conductance was calculated by dividing the mean blood perfusion by the MAP at each timepoint.

### 2.7 Immunohistochemistry

Knee joint afferents were labeled using the retrograde fluorescent dye, Fluoro-Gold. Male rats (*n* = 4 per group) were deeply anaesthetized (2–4% isoflurane; 100% oxygen at 1 L/min) 5 days prior to experimental endpoint and 10 µL of Fluoro-Gold (2% solution in saline; Fluorochrome, CO, United States) was injected into the ipsilateral knee joint. Five days following Fluoro-Gold administration, anaesthetized animals were transcardially perfused with saline followed by 4% paraformaldehyde. DRGs from L3 were removed, cryopreserved with sucrose, embedded in OCT (Sakura Finetek, CA, United States) and stored at −20°C until sectioning. The DRGs were sectioned at 12 µm and mounted onto slides which contained 5 non-consecutive sections.

DRG sections were treated with rabbit-anti-PAR4 (1:250, catalogue number: AB137927 Abcam, Cambridge, United Kingdom) primary antibody followed by Alexa fluor 488 goat-anti-rabbit (1:500, Invitrogen, Carlsbad, CA, United States) secondary antibody. Omission of the primary antibody in some sections acted as a control. Slides were viewed under a Ziess Axio Imager 2 (Zeiss, Oberkochen, Germany) at 10x magnification. Photomicrographs were taken using an AxioCam HRm camera (Zeiss, Oberkochen, Germany) and analyzed offline using ImageJ software. The total number of Fluoro-Gold-positive neurones were counted and the percentage that expressed PAR4 was calculated.

### 2.8 Saphenous Nerve Histology

Saphenous nerves were harvested proximal to the ipsilateral knee joint of early and late phase MMT and MIA animals and processed as previously described (Philpott et al., 2017). The nerve was sectioned at a thickness of 100 nm and placed on a copper grid and inserted into a JEOL JEM 1230 transmission electron microscope (JEOL Corp. LTd., Tokyo, Japan) for visualisation. One nerve cross-section from each animal was visually divided into nine quadrants and three photomicrographs from quadrants one, five, and nine were taken so as to sample equally across the nerve. Images were taken at a magnification of 2,500X. Axons were visually inspected to identify those with compact, uniform myelin which were deemed non-damaged, and those with disrupted or extruding myelin which were classified as damaged axons (O'Brien and McDougall, 2020). The percentage of axons that were damaged in each nerve was calculated. Myelin thickness of both small diameter (<3 μm) and large diameter (>3 μm) fibres was measured by calculating the G-ratio. The following equation was used: G=aA where “a” is the internal axonal area and “A” is the external axonal area.

### 2.9 Serum Levels of Inflammatory Mediators

Intra-cardiac blood samples were taken from naïve, late phase, and early phase OA rats and serum was subsequently isolated and stored at −20 °C. For ELISA analysis, serum samples were thawed, aliquoted (25 μL), and the concentrations of five cytokines (IL-1β, IL-10, Il-17A, IL-6, and TNF-α) were measured using a Luminex microbead-based suspension array (Invitrogen, Carlsbad, CA, United States) following the manufacturer’s protocol in a 96 well plate format. The plate was read using a Bio-Plex 200 system (Bio-Rad, Hercules, California, United States) and analyzed using Bio-Plex Manager 6.0 software (Bio-Rad, Hercules, CA, United States).

### 2.10 Drugs

Urethane, sodium monoiodoacetate, and rhodamine 6G were obtained from Sigma Aldrich (St. Louis, MO, United States) and were dissolved in 0.9% saline. Pepducin P4pal10 was synthesized by Genscript (Piscataway, NY, United States) and also dissolved in 0.9% saline. Physiological buffer (135 mM NaCl, 20 mM NaHCO3, 5mM KCl, 1 mM MgSO4-7H2O, pH = 7.4) was prepared in the lab.

### 2.11 Statistical Analysis

All data were expressed as mean ± SEM. Data were tested for Gaussian distribution by examining the residuals in QQ plots. Spontaneous firing data is non-parametric and was analyzed by estimating the difference between proportions. Data from multiplex, immunohistochemistical, g-ratio, behavioural pain, and the electrophysiological experiments were normally distributed and were therefore analyzed using parametric statistics (2-way analysis of variance (ANOVA), 1-way ANOVA, or unpaired 2-tailed Student’s *t* test). A *p* value less than 0.05 was considered statistically significant.

## 3 Results

### 3.1 Myelin Thickness and Nerve Damage in Osteoarthritis Models

**FIGURE 2:**
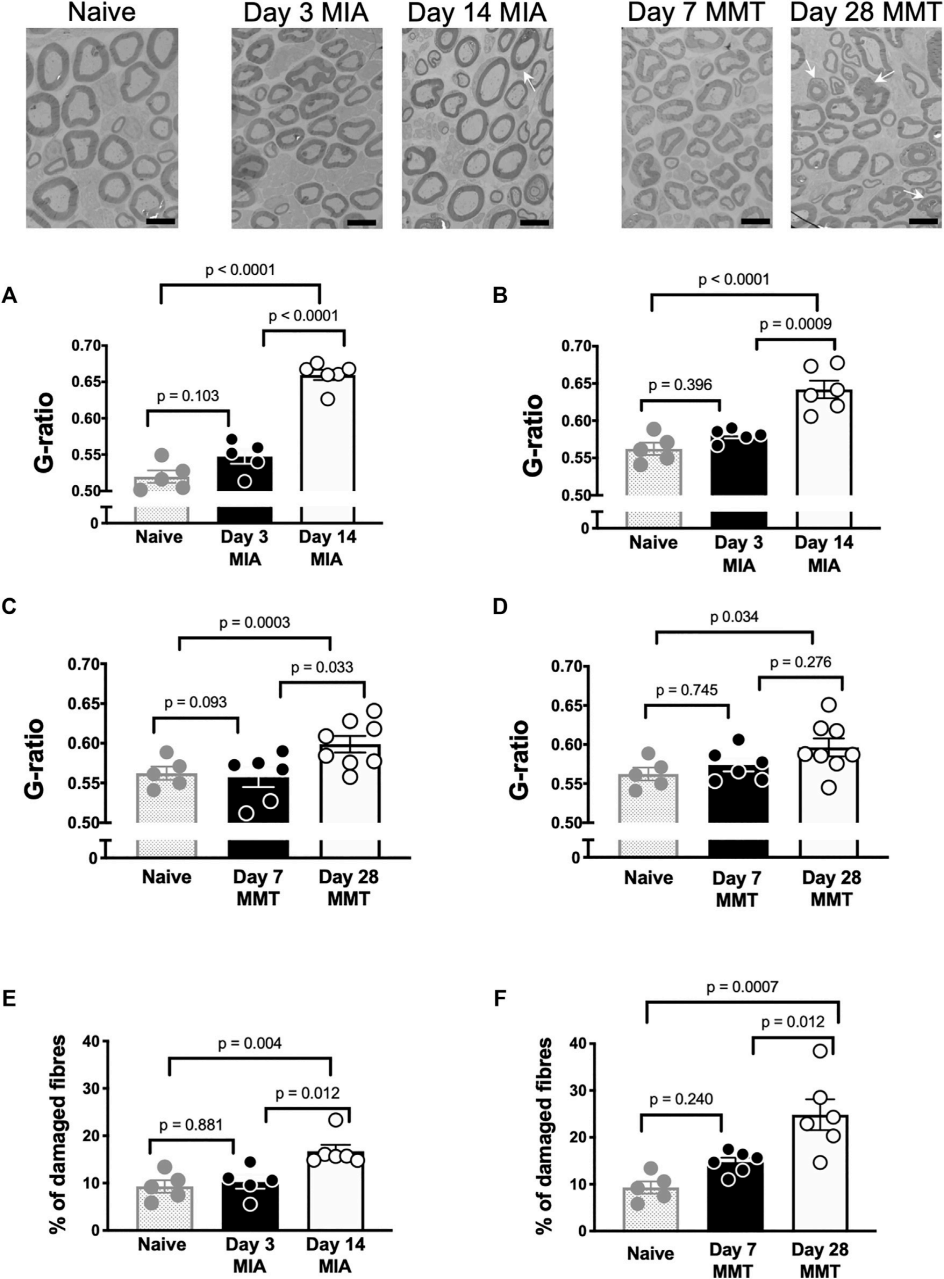
Joint Nerve Damage in the MIA and MMT models of Arthritis. Representative electron micrographs of the saphenous nerve from naïve, MIA, and MMT animals. G-ratio values were significantly different when comparing the day 14 timepoint in the MIA model with both naïve and day 3 MIA animals; both in small diameter axons **(A)** and large diameter axons **(B)** (*p* < 0.05, *n* = 5–6, 1-way ANOVA). G-ratio values did not significantly differ between day 7 and day 28 MMT for small **(C)** or large **(D)** diameter axons (*p* > 0.05, *n* = 5–6, 1-way ANOVA). The percentage of damaged axons (arrows) was significantly higher in day 14 MIA animals compared to naïve and day 3 MIA **(E)** (*p* < 0.01, *n* = 5–6, 1-way ANOVA). The percentage of damaged axons (arrows) was also significantly higher in day 28 MMT animals compared to naïve and day 7 animals **(F)** (*p* < 0.05, *n* = 5–6, 1-way ANOVA). Data presented as means ± SEM. Arrows indicate damaged axons, scale bar = 6 μm.

Saphenous nerves were harvested for histological assessment of demyelination and overall nerve damage (Figure 2). G-ratio analysis revealed significant loss of myelin in both small and large diameter axons at day 14 post MIA administration compared to naïve and day 3 MIA (Figures 2A–B, *p* < 0.05, students t-test, *n* = 5–6). The percentage of nerves with abnormal morphology was also enhanced at day 14 with 17 ± 1% exhibiting damage compared to 10 ± 1% on day 3 MIA and 9.3 ± 0.3% in naïve animals (Figure 2E, *p* < 0.01, *n* = 5–6). Axons appeared morphologically similar in day 3 MIA and naïve animals (Figure 2E, *p* > 0.05, *n* = 5–6). The MMT model also caused demyelination at the late phase of the model but not at day 7 (Figures 2C–D, *p* > 0.05, *n* = 6–7). Comparison of axon morphology between early and late stage MMT and naïve animals did reveal, however, that there was a significant increase in abnormal morphology at the later timepoint with 29 ± 5% of fibres exhibiting axonal damage compared to 14 ± 1% at day 7 and 9.3 ± 0.3% in naïve animals (Figure 2F, *p* < 0.05, *n* = 6–7).

### 3.2 Neuronal PAR4 Expression

**FIGURE 3:**
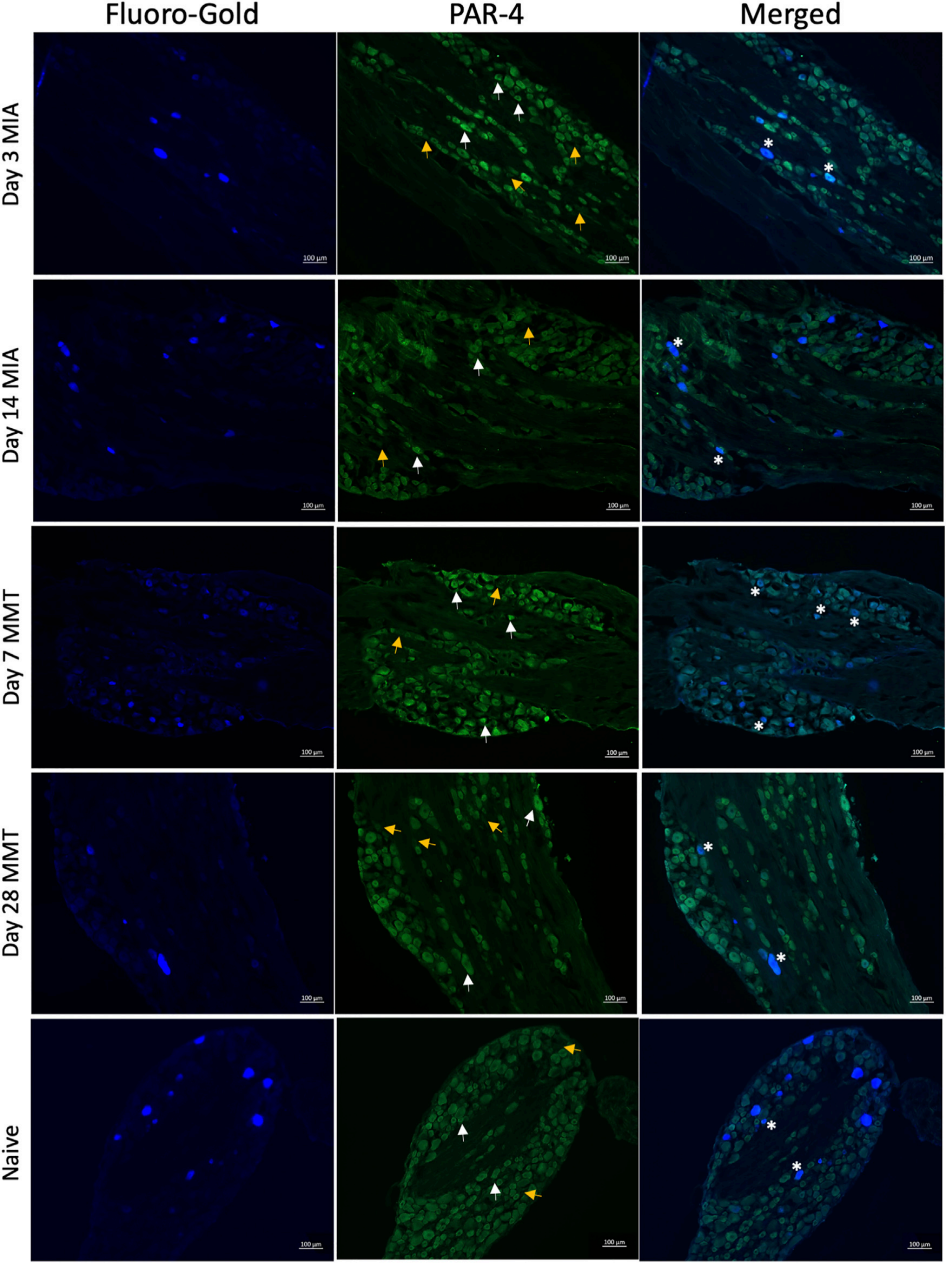
PAR4 Expression in Joint DRG Neurons. Representative photomicrographs of Fluoro-gold (FG) traced joint neurons that PAR4. White arrows indicate positive expression of PAR4 and yellow arrows indicate a lack of expression, asterisks represent coexpression.

**FIGURE 4:**
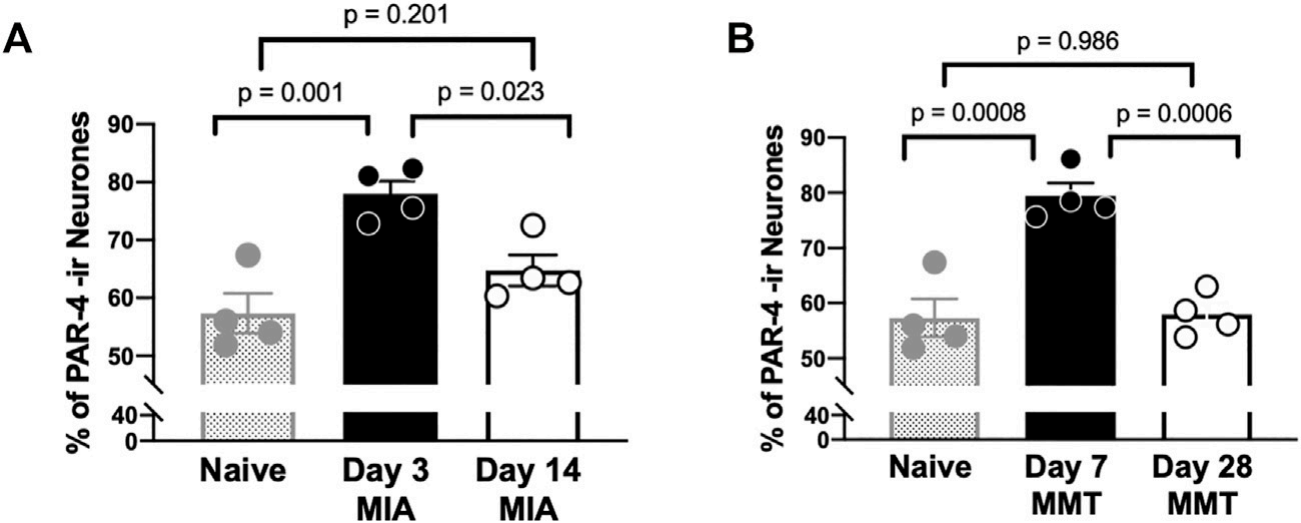
PAR4 Expression is Elevated in Early OA/PTOA. The percentage of PAR4 expressing joint neurons was significantly higher in day 3 MIA compared to day 14 **(A)** (*p* < 0.01, student’s *t*-test, *n* = 4 animals per group). Similarly, higher levels of PAR4 were observed 7 days following MMT surgery compared to Day 28 **(B)** (*p* < 0.001, student’s *t*-test, *n* = 4 animals per group). Data are means ± SEM.

Immunohistochemical analysis of PAR4 expression in Fluoro-gold positive DRG neurones revealed increased expression in both the MIA and MMT models at early timepoints compared to naïve and end-stage OA animals (Figures 3, 4). In naïve animals, 57 ± 3% of fluoro-gold positive neurones co-expressed PAR4. Three days following MIA administration, 78 ± 2% of joint neurones expressed PAR4 compared to 65 ± 3% at the 2 week timepoint (Figure 4A, *p* < 0.05, 1-way ANOVA, *n* = 4). A similar level of expression was observed in the MMT model where 7 days following surgery 79 ± 2% of fluoro-gold positive neurones co-expressed PAR4 compared to only 58 ± 2% on day 28 of the model (Figure 4B, *p* < 0.001, *n* = 4). PAR4 was expressed in similar size neurones across all of the different cohorts (Table 1).

**TABLE 1:** Comparison of PAR4 Expression by Neuronal cell size. The distribution of PAR4 expressing fluoro-gold dorsal root ganglia was quanitified based on cell diameter. Similar levels of PAR4 were observed within each model regardless of neuronal size. *p < 0.05 denotes statistical significance (2-way ANOVA) day 7MMT versus day 28 MMT. Data presented as means ± SEM. ND, not detected.

% Joint Afferents Expressing PAR4					
Cell diameter (μm)	Naive	Day 3 MIA	Day 14 MIA	Day 7 MMT	Day 28 MMT
10.00–19.99	59.9 ± 0.2	69.1 ± 0.1	63.0 ± 0.04	73.9 ± 0.1	72.7 ± 0.1
20.00–29.99	46.3 ± 0.1	70.7 ± 0.03	57.9 ± 0.06	68.6 ± 0.07	50.5 ± 0.05
30.00–39.99	65.2 ± 0.1	75.0 ± 0.03	69.2 ± 0.05	89.4 ± 0.02*	55.6 ± 0.08
40.00–49.99	66.1 ± 0.1	90.1 ± 0.04	69.1 ± 0.04	93.8 ± 0.06	80.4 ± 0.07
50.00–59.99	41.7 ± 0.4	76.2 ± 0.01	62.5 ± 0.4	100.0	50.0
60.00–69.99	ND	ND	ND	50.0	ND
70.00–79.99	ND	100.0	ND	0.00	ND

### 3.3 Blockade of PAR4 Reduced Joint Nociceptor Firing at Early Timepoints

Joint afferent fibres were characterized based on their mechanical thresholds, baseline evoked firing rates, spontaneous activity, conduction velocity, and electrical thresholds (Summarized in Table 2). Units with a conduction velocity <2 m/s were considered type IV afferents and units with a conduction velocity >2 m/s were considered type III. Joint afferents from day 3 MIA animals fired at a significantly higher rate compared to day 14 MIA joints (*p* < 0.05, Student’s *t*-test, *n* = 10–11). The proportion of joint afferents that were spontaneously active in day 3 MIA animals was 73% compared to 50% of fibres in day 14 animals. This difference, however, was not statistically significant (*p* > 0.05, *n* = 10–11, difference in proportions).

**TABLE 2:** Electrophysiological characteristics of joint afferents from MIA and MMT animals. Fibres recorded from MIA and MMT animals during early and established OA were characterised based on baseline mechanical thresholds and evoked firing frequency, the presence spontaneous firing, electrical thresholds, and the calculated conduction velocity. *p < 0.05 denotes statistical significance comparing the two timepoints in each model using a Student’s *t*-test. Data presented as means ± SEM and (range). ND, not determined.

Fibre type	Mechanical threshold (mNm)	Evoked baseline firing (action potentials/rotation)	Spontaneous fibres (%)	Electrical threshold (V)	Conduction velocity (m/s)	*n*
MIA						
Day 3	10 ± 1 (4–19)	65 ± 8 (25–112)*	73	3 ± 0.1 (2.5–3.0)	1.6 ± 0.9 (0.61–2.18)	11
Day 14	16 ± 3 (2–30)	34 ± 6 (11–70)	50	3 ± 0.4 (2.0–4.0)	3.1 ± 1.2 (1.85–4.66)	10
MMT						
Day 7	11 ± 2 (4–25)	35 ± 5 (13–75)	56	3 ± 0.4 (2.5–3.75)	2.3 ± 0.8 (1.60–3.63)	16
Day 28	10 ± 2 (3–25)	38 ± 5 (13–83)	57	3.5 ± 0.4 (3.0–4.0)	2.6 ± 1.4 (0.66–4.24)	14

In MMT animals, fibres from the early and late timepoints fired at similar rates in response to noxious rotation of the joint. Comparable spontaneous firing was also observed in early and late stage MMT joints: 56% of day 7 MMT fibres, and 57% of day 28 MMT fibres (*p* > 0.05, *n* = 14–16, difference in proportions). There were no significant differences in mechanical or electrical thresholds, or conduction velocities between any of the animal groups (Table 2).

**FIGURE 5:**
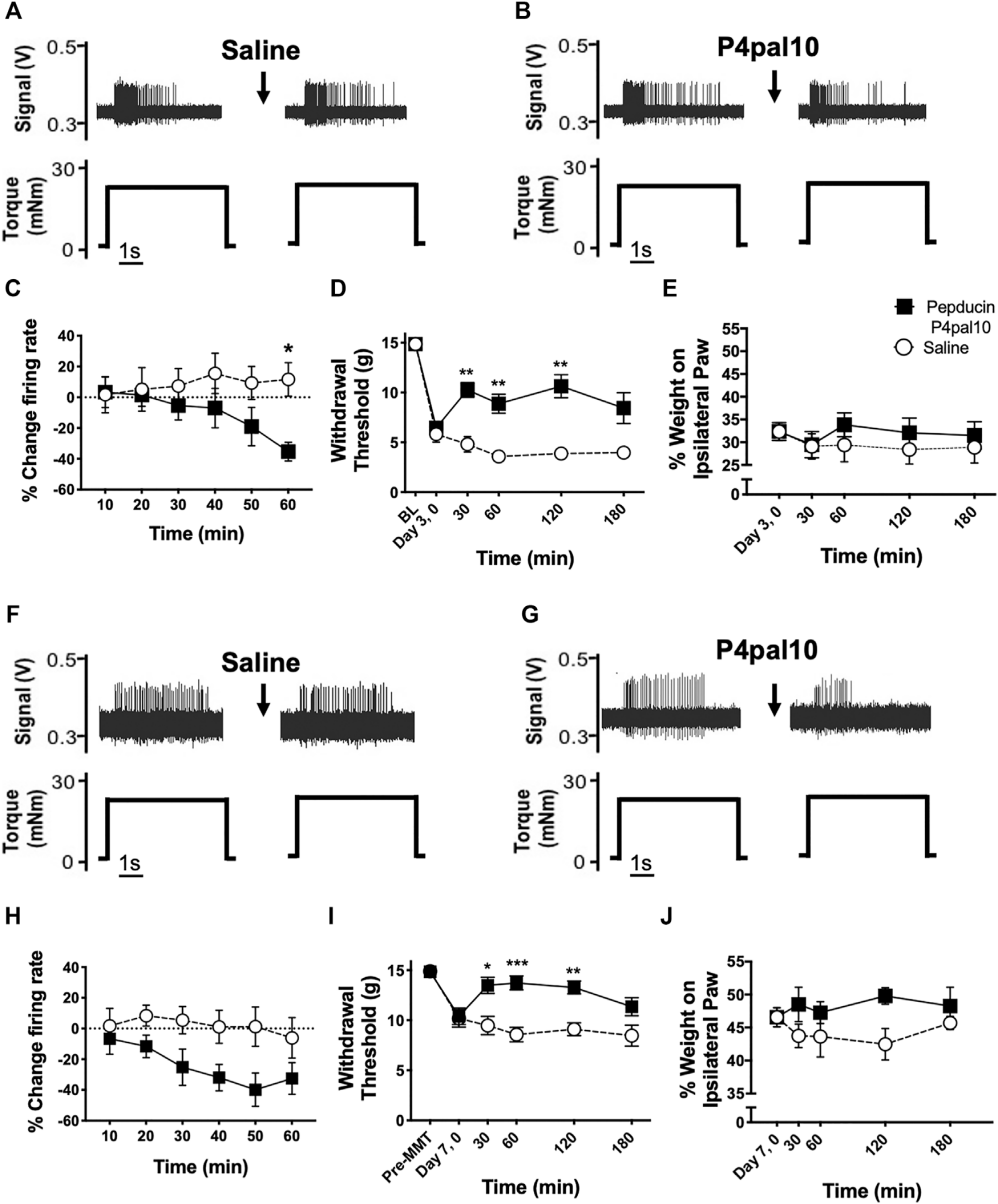
Effect of PAR4 blockade on Early OA/PTOA Pain Behaviour and Joint Electrophysiology. Representative traces of knee joint afferent recordings from day 3 MIA animals **(A–B)** and day 7 MMT animals **(F–G)** before and after administration of saline or pepducin P4pal10. Evoked firing is in response to noxious rotation of the knee joint. Systemic administration of pepducin P4pal10 reduced afferent firing relative to baseline over a 60 min timecourse in both day 3 MIA **(C)** and day 7 MMT **(H)** joint afferents (*n* = 8–10 fibres per group, 2-way RM-ANOVA). Administration of pepducin P4pal10 improved withdrawal thresholds for 120 min compared to saline vehicle in day 3 MIA animals **(D)** (*p* < 0.001, 2-way RM-ANOVA, *n* = 7). Weight bearing deficits were not improved following drug administration **(E)** (*p* > 0.05, 2-way RM-ANOVA, *n* = 7). When day 7 MMT animals were treated with pepducin P4pal10, both secondary allodynia **(I)** and weight bearing deficits **(J)** were improved (*p* < 0.05–0.001, 2-way RM-ANOVA, *n* = 7). *p < 0.05, **p < 0.01 ***p < 0.001, ****p < 0.0001 denotes statistical significance using *post hoc* Sidak’s Multiple Comparisons Test. Data presented as means ± SEM.

Examples of electrophysiological traces of joint afferent fibres before and after administration of saline or pepducin P4pal10 in day 3 MIA and day 7 MMT animals are represented in Figure 5. Pepducin P4pal10 significantly reduced joint afferent firing in response to noxious rotation of the joint in day 3 MIA rats (Figure 5E; *p* < 0.05, *n* = 8, 2-way RM-ANOVA) with a maximal reduction of 35 ± 6% compared to baseline firing, 60 min following drug administration. PAR4 blockade also significantly reduced nociceptor firing in day 7 MMT animals (Figure 5J; *p* < 0.05, *n* = 8–10, 2-way RM-ANOVA) where at 50 minutes a maximum of 40 ± 10% reduction in firing rate was achieved.

### 3.4 Effect of PAR4 Blockade on Pain Behavior in Early Onset Osteoarthritis

Day 3 MIA model animals presented with secondary allodynia (Figure 5C) which was partially reversed following treatment with pepducin P4pal10 (*p* < 0.0001, *n* = 7, Figure 5C). Animals also exhibited hindlimb weight bearing deficits; however, pepducin P4pal10 did not alter these deficits when compared to saline vehicle treatment (Figure 5D, *p* > 0.05, *n* = 7).

Seven days post MMT surgery, animals also exhibited reduced paw withdrawal thresholds (Figure 5H) and mild weight bearing deficits (Figure 5I). In this model, treatment with pepducin P4pal10 was effective in both pain behaviour tests. Mechanical allodynia was reduced compared to vehicle for 120 min (Figure 5H, *p* < 0.0001, *n* = 8), and the weight bearing differential also improved (Figure 5I, *p* < 0.01, *n* = 8).

### 3.5 PAR4 Antagonism and Joint Nociceptor Firing in End-Stage Osteoarthritis

**FIGURE 6:**
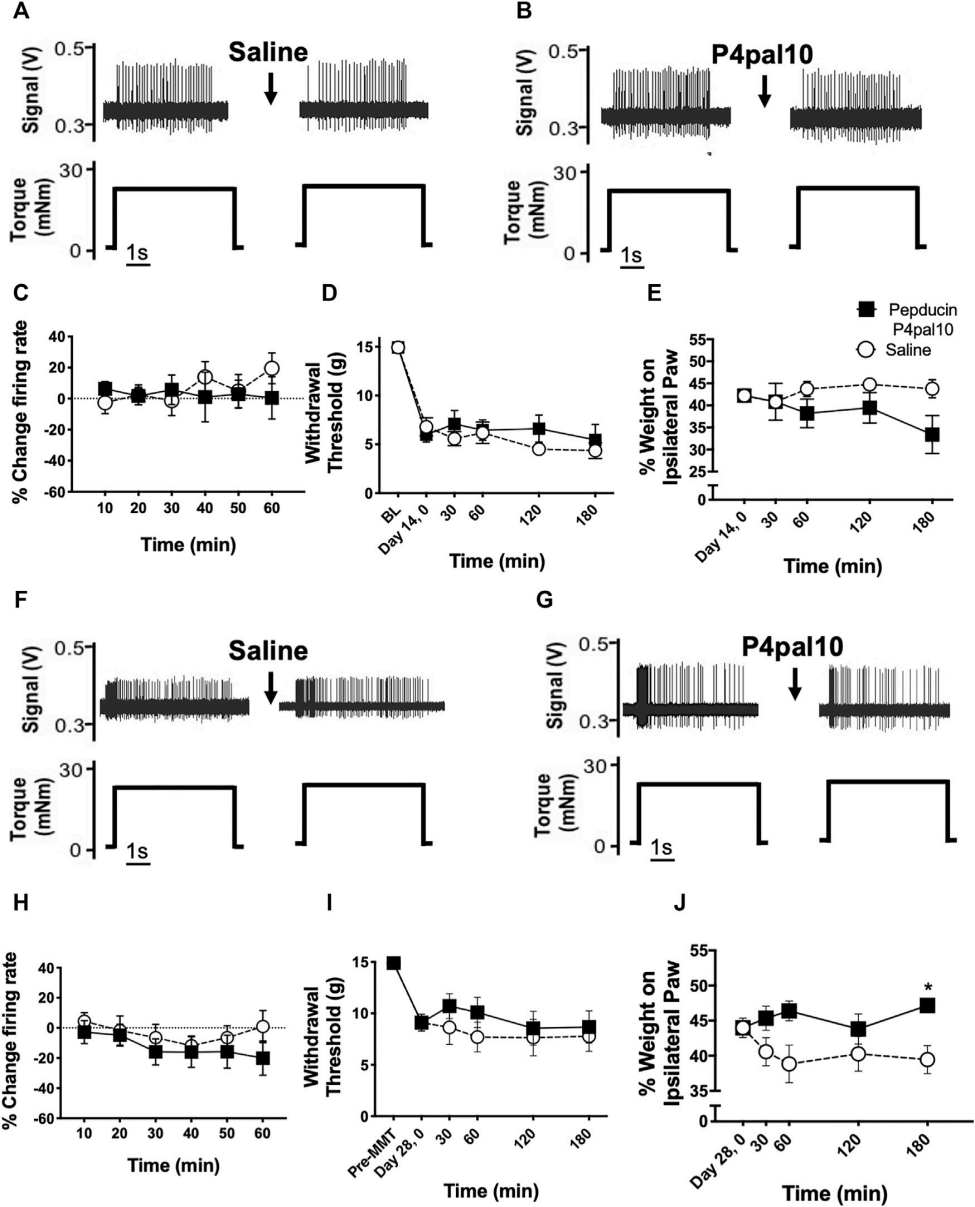
Effect of PAR4 blockade on Endstage OA/PTOA Pain Behaviour and Joint Electrophysiology. Representative traces of knee joint afferent recordings from day 14 MIA animals **(A–B)** and day 28 MMT animals **(F–G)** before and after administration of saline or pepducin p4pal10. Blockade of PAR4 with pepducin P4pal10 failed to reduce joint afferent firing in day 14 MIA animals **(C)** or day 28 MMT animals **(H)** compared to saline vehicle (*p* > 0.05, 2-way RM-ANOVA, *n* = 7–11). Administration of pepducin P4pal10did not significantly improve secondary allodynia **(D)** or weight bearing deficits **(E)** in day 14 MIA animals (*p* > 0.05, 2-way RM-ANOVA, *n* = 7). When day 28 MMT animals were treated with pepducin p4pal10, it did not improve withdrawal threshold **(I)** (*p* > 0.05, 2-way RM-ANOVA, *n* = 7) but did significantly reduce hindlimb weight bearing deficits at 180 min **(J)** (*p* < 0.05, 2-way RM-ANOVA, *n* = 7). *p < 0.05 denotes statistical significance using *post hoc* Sidak’s Multiple Comparisons Test. Data presented as means ± SEM.

Examples of electrophysiological traces of joint afferent fibres before and after administration of saline or pepducin P4pal10 in day 14 MIA and day 28 MMT animals are represented in Figure 6. Single unit recording from joint mechanoreceptors in day 14 MIA animals revealed that blockade of PAR4 had no effect on joint afferent firing over the 60 min time course (Figure 6E; *p* > 0.05, *n* = 7–9). A similar lack of effect was observed in day 28 MMT animals, where pepducin P4pal10 did not elicit a significant change in nociceptor activity (Figure 6J; *p* > 0.05, *n* = 10–11).

### 3.6 PAR4 Antagonism and Pain Behavior in End-Stage Osteoarthritis

Fourteen days after MIA induction, animals had similar levels of hindlimb mechanosensitivity as day 3 MIA rats (day 14: 6 ± 0.5 g, day 3: 6 ± 0.6 g withdrawal threshold); however, weight bearing deficits were less severe. At this end-stage phase of the disease, animals bore 42 ± 1% weight on the ipsilateral hindpaw compared to 32 ± 2% on day 3 (*p* < 0.001, *n* = 14–15).

When compared to vehicle control, pepducin P4pal10 had no effect on withdrawal threshold or hindlimb weight bearing in day 14 MIA animals (Figures 6C,D, *p* > 0.05, *n* = 7).

28 days following MMT surgery, the referred pain and weight bearing deficits observed on day 7 persisted were not significantly different from the earlier timepoint (*p* > 0.05, *n* = 12–16). Pepducin P4pal10 had no effect on von Frey hair mechanosensitivity (Figure 6H, *p* > 0.05, *n* = 8), but did significantly improve hindlimb incapacitance compared to vehicle control (Figure 6I, *p* < 0.05, *n* = 8).

### 3.7 Serum Cytokine Levels in the MIA and MMT Models

Serum cytokines were measured to gauge the degree of inflammation in naïve animals and at early and late timepoints in the MIA and MMT models (Table 3). In MIA animals, levels of the pro-inflammatory mediators IL-6 and TNF-α were higher at day 3 compared to day 14 (*p* < 0.05, *n* = 7). The anti-inflammatory cytokine IL-10 was also significantly elevated at the earlier timepoint (*p* < 0.05, *n* = 7). Both IL-10 and TNF-α levels were also significantly elevated in day 3 MIA when compared to naïve animals. Serum concentration of IL-1β was below the limit of detection in the samples from MIA animals.

**TABLE 3:** Measurement of serum cytokines in MIA and MMT animals. Serum cytokines IL-10, IL-1β, IL-17A, IL-6 and TNF-α were quantified in naïve animals and during early and late timepoints in MIA and MMT animals. In both models, several cytokines were evelated during the early OA when compared to the late timepoint. *p < 0.05 denotes statistical significance comparing the two timepoints within the same model using a 1-way ANOVA. ##*p* < 0.01, #*p* < 0.05 compared to naïve control. Data presented as means ± SEM. ND, not determined.

	IL-10	IL-1β	IL-17A	IL-6	TNF-α	n
Naive						
	319.5 2 ± 122.9	ND	ND	ND	61.44 ± 24.0	6
MIA						
Day 3	1,377 ± 301.7*##	ND	257.3 ± 32.1	33.8 ± 6.3*	360.8 ± 106*#	7
Day 14	625.2 ± 88.9	ND	162.4 ± 31.5	12.6 ± 3.1	114.4 ± 29.3	7
MMT						
Day 7	329.2 ± 48.3	703.5 ± 110.5*	37.29 ± 4.1*	78.65 ± 7.6	ND	5
Day 28	188.1 ± 48.0	252.9 ± 77.3	15.3 ± 5.7	53.69 ± 14.6	ND	5

In the MMT model, serum levels of IL-1β and IL-17A were elevated at day 7 compared to day 28 (*p* < 0.05, *n* = 5). These molecules were not detectable in naïve animals so no comparison could be made. There were no significant differences in the amount of serum IL-10 or IL-6 throughout the development of this model (*p* > 0.05, *n* = 5). The levels of TNF-α were below the limit of detection in most of the samples from MMT animals.

### 3.8 Effect of PAR4-Inhibition on MIA and MMT-Induced Joint Inflammation

**FIGURE 7:**
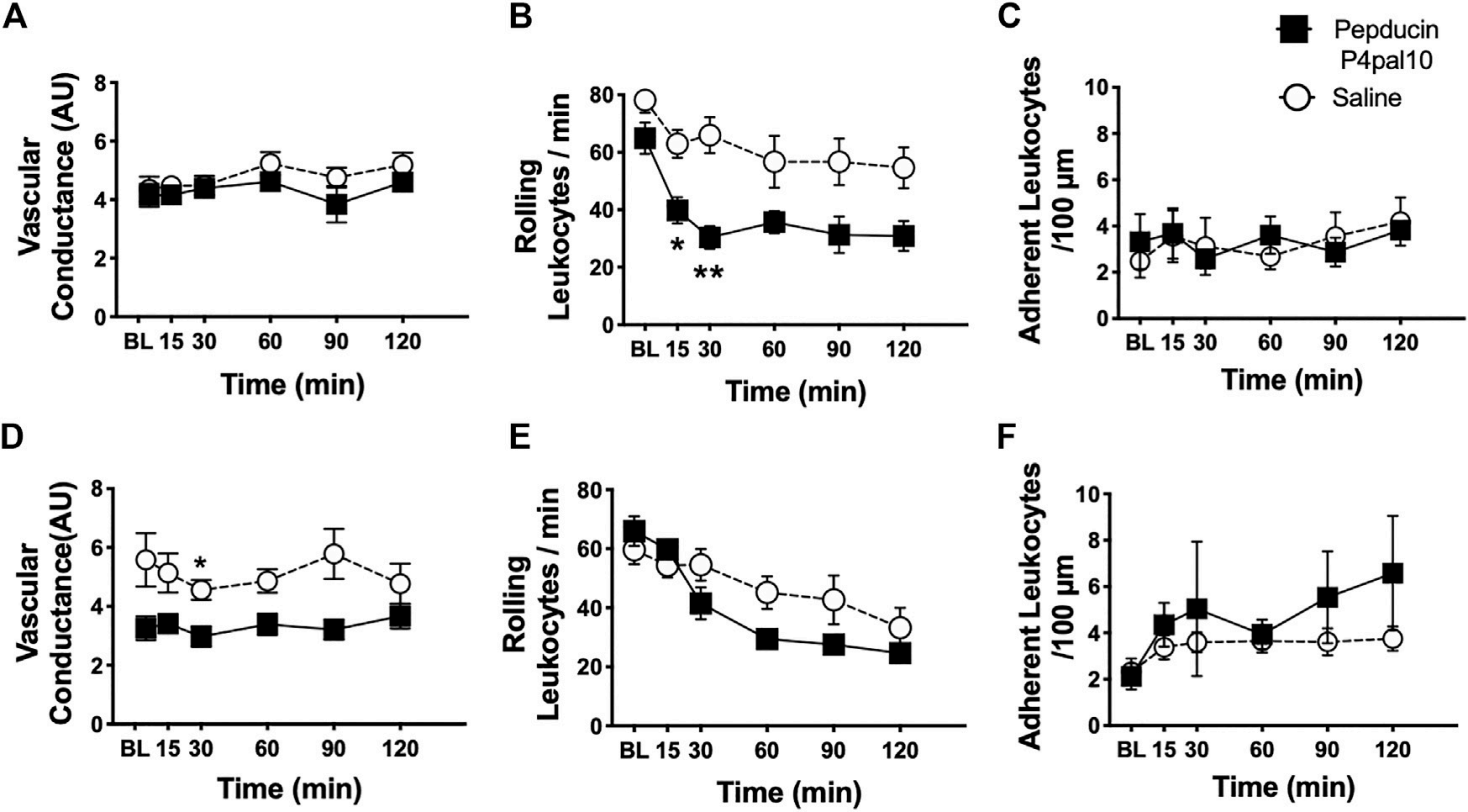
Effect of PAR4 Blockade on Joint Inflammation in Early OA/PTOA. Administration of pepducin P4pal10 failed to improve vascular conductance **(A)** (*p* > 0.05, 2-way RM-ANOVA, *n* = 6) but significantly reduced rolling leukocytes in day 3 MIA animals **(B)**(*p* < 0.05, 2-way RM-ANOVA, *n* = 6). No significant effect was observed on adherent leukocyte trafficking in the same animals **(C)** (*p* > 0.05, 2-way RM-ANOVA, *n* = 6). PAR4 blockade in day 7 MMT animals did induce significant reductions in both vascular conductance **(D)** and rolling leukocytes **(E)** (*p* < 0.05, 2-way RM-ANOVA, *n* = 9–12) but failed to inhibit adherent leukocyte movement **(F)** (*p* > 0.05, 2-way RM-ANOVA, *n* = 9–12). *p < 0.05, **p < 0.01 denotes statistical significance using *post hoc* Sidak’s Multiple Comparisons Test. Data presented as means ± SEM.

Joint inflammation was assessed at both timepoints in the two OA models; however, as described elsewhere (Guzman et al., 2003; Orita et al., 2011) inflammation was not present in the later stages of the disease (data not shown). When administered on day 3 of the MIA model, pepducin P4pal10 had no effect on synovial vascular conductance (Figure 7A, *p* > 0.05, *n* = 6). Intravital microscopy revealed that the number of rolling leukocytes was significantly reduced following PAR4 blockade compared to vehicle treatment (Figure 7B, *p* < 0.05, *n* = 6). There was no effect of pepducin P4pal10 on leukocyte adherence (Figure 7C, *p* > 0.05, *n* = 6).

PAR4 blockade in Day 7 MMT knee joints significantly reduced joint vascular conductance over a 2 h period (Figure 7D, *p* < 0.05, *n* = 9–12). Pepducin P4pal10 treatment had no effect on either rolling or adherent leukocytes (Figure 7E, *p* > 0.05, *n* = 9–12) when compared to vehicle.

## 4 Discussion

Despite a growing body of evidence implicating serine proteinases and their cognate receptors, PARs, in arthritis pathophysiology and pain, very little is known regarding the role of PAR4 in OA. The data presented here demonstrate that PAR4 is involved in early onset OA pain when joint inflammation is present. Conversely, PAR4 does not appear to be a relevant target in end-stage OA when inflammation has resolved and the pain is more nociceptive and neuropathic.

Chronological characterisation of the MIA and MMT models of OA have revealed distinct time-dependent phenotypes with an early pro-inflammatory phase while endstage disease coincided with periperhal neuropathy. Inflammatory markers including TNFα and IL-6 were elevated on day 3 of the MIA model whereas IL-1β and IL-17A were increased in the serum of day 7 MMT animals. These pro-inflammatory cytokines declined in the later stages of OA. Analysis of saphenous nerves demonstrated demyelination and irregular axon morphology in the late phase of the MIA model compared to the early phase. In contrast, nerves from MMT animals did not undergo demyelination although significantly more axons were damaged in late MMT compared to the early timepoint. These findings corroborate other studies that have demonstrated an early inflammatory component in the MIA model including synovitis, elevated inflammatory cytokines, and pain that could be attenuated with diclofenac but not gabapentin (Fernihough et al., 2004; Ivanavicius et al., 2007; Orita et al., 2011). From day 14 onwards in the MIA model, the expression of the nerve damage marker ATF-3 in DRG neurones is enhanced, joint cytokine levels dissipate, and pain is reversed with gabapentin while the NSAIDs diclofenac and celecoxib are ineffective (Fernihough et al., 2004; Orita et al., 2011). In contrast Bove et al. (2006), found that treatment of MMT joints on day 21 with gabapentin or celecoxib were both analgesic; however, gabapentin was more efficacious (Bove et al., 2006). Amitriptyline also attenuated pain behaviour and joint afferent firing in day 28 MMT animals, further supporting a neuropathic component in endstage disease (O'Brien and McDougall, 2020).

Previous studies demonstrated that activation of articular PAR4 mediates inflammatory-pain (McDougall et al., 2009; Russell et al., 2010; Russell et al., 2011; McDougall, 2012) while its role in neuropathic-pain is unknown. Here, treatment with a PAR4 antagonist during the acute inflammatory phase of MIA and MMT reduced joint nociceptor firing and improved secondary allodynia. PAR4 blockade during endstage neuropathic OA, however, failed to alter pain behaviour or joint afferent firing in either model. Local administration of the synthetic PAR4 agonist AYPGKF-NH2 to the rat knee joint has previously been shown to cause peripheral sensitization via a bradykinin B2 receptor mechanism (Russell et al., 2010). Elsewhere, Cialdai and colleagues found that bradykinin levels are elevated at day 3 in the MIA model (Cialdai et al., 2009), which could in part be a consequence of enhanced PAR4 activity at this early timepoint. Since bradykinin also increases joint nociceptor activity (Neugebauer et al., 1989), an escalation in the PAR4-bradykinin pathway could be responsible for the anti-nociceptive effect of pepducin P4pal10 in the early phase of the OA models described here.

It has been previously shown that inflammatory mediators modulate PAR expression in joints (Xiang et al., 2006). In the present study, circulating pro-inflammatory cytokine levels were elevated in early but not late OA animals. Furthermore, the expression of PAR4 in joint DRG neurones was similarly enhanced during the early inflammatory phase of both arthritis models compared to the later timepoints. Thus, increased PAR4 expression correlated with timepoints at which the joints were inflamed and provides an explanation as to why PAR4 was most effective in early OA.

The current study also examined the role of PAR4 in early OA joint inflammation. Previously, PAR4 activation with a peptide agonist has been found to cause synovial hyperaemia and oedema in the knee which could be blocked by P4pal10 (McDougall et al., 2009). These pro-inflammatory effects of PAR4 were again mediated by bradykinin being released from connective tissue mast cells (Russell et al., 2011). In the OA models described here, blocking PAR4 had a mild anti-inflammatory effect in day 3 MIA and day 7 MMT animals. In contrast to these early timepoints, there was minimal leukocyte trafficking in day 14 MIA and day 28 MMT animals (data not shown) and as such modulation of PAR4 was not possible due to the lack of joint inflammation. Taken together, it appears that PAR4 is exclusively associated with the acute inflammatory phase of OA but has little relevance in end stage disease.

In summary, the present investigation confirmed a transient inflammatory response in the early stages of OA in the MIA and MMT models. During this phase, joint afferent PAR4 expression was elevated and blockade of the receptor ameliorated peripheral sensitization and pain. During the post-inflammatory endstage of the models, joint nerve integrity deteriorated and PAR4 blockade was ineffective at reducing pain. Taken together, PAR4 seems to be involved in acute inflammatory OA pain but not chronic neuropathic OA pain. Blockade of PAR4 may therefore be most suitable for early OA pain treatment or during episodic flares.

## Data Availability

The raw data supporting the conclusion of this article will be made available by the authors, without undue reservation.
